# I can't get no satisfaction: burnout, stress, and depression in Latin medical students

**DOI:** 10.47626/2237-6089-2024-0818

**Published:** 2025-12-05

**Authors:** Madeleine Morris, Luiza Palmieri Serrano, Krina Patel, Jorge Cervantes

**Affiliations:** 1 Vanderbilt University Medical Center Department of Psychiatry and Behavioral Sciences Nashvillle TN USA Department of Psychiatry and Behavioral Sciences, Vanderbilt University Medical Center, Nashvillle, TN, USA.; 2 Nova Southeastern University Kiran C. Patel College of Allopathic Medicine Fort Lauderdale FL USA Kiran C. Patel College of Allopathic Medicine, Nova Southeastern University, Fort Lauderdale, FL, USA.

**Keywords:** Burnout, medical students, Latin America

## Abstract

The state of emotional, physical, and mental exhaustion caused by excessive and prolonged stress, known as burnout syndrome (BS), is not only affecting the medical workforce but medical students in training. Gender, race, ethnicity, and potentially other variables can serve as significant risk factors contributing to BS among medical students. Despite the importance of understanding these disparities, very few studies in the U.S. have analyzed race or ethnicity amongst their cohorts. However, there exists extensive information on burnout in students from Latin America, which serves as the primary focus of this review. A systematic literature search was conducted using pertinent terms in English and Spanish. Our review found that the prevalence of BS in Latin American countries varies widely, ranging from 4.3 to 43.90% pre-COVID-19 pandemic. Variability in the educational environment and the complex interplay of cultural, academic, and systemic factors appear to contributing to burnout among students. Post-pandemic investigations reveal even higher prevalences, particularly among women. High rates of depression and anxiety are also reported during the COVID-19 pandemic. The reviewed data showed that BS can become further exacerbated and complicated by existing psychiatric comorbidities amongst Latin American medical students. It is possible that we may observe continued upward trajectories in burnout trends among both healthcare workers and medical students in this post-COVID-19 pandemic era. These insights call for tailored interventions addressing not only burnout but also the interconnected mental health challenges faced by medical students in Latin America.

## Burnout in medical students

Burnout is characterized as a state of emotional, physical, and mental exhaustion caused by excessive and prolonged stress.^1^ These challenges can manifest in three dimensions: emotional exhaustion, depersonalization in their interactions with others, and reduced feelings of personal accomplishment.^2^ Burnout syndrome (BS) is common among individuals who experience a decline in their daily functioning due to the demanding psychological requirements in their workplace. Over the last few decades, extensive research has been conducted within the medical profession. Among medical residents, burnout has rates of up to 76%.^3^ Physician burnout has been identified as a key factor correlated with a range of significant issues in healthcare, including occurrences of medical errors, diminished patient satisfaction, and extended recovery periods.^4^

In recent years, there has been a growing focus on burnout during medical training. The literature reveals that burnout has been prevalent during medical school, even in the pre-COVID-19 pandemic era, with major multi-institutional studies in the United States estimating that a minimum of half of all medical students may experience burnout during their medical education.^5-9^ A more recent meta-analysis calculates a prevalence of burnout of 49.99% for the U.S. population.^10^

Medical students often deal with issues such as sleep deprivation, limited control over their schedules, and diminished self-confidence.^11^ Research indicates that those who encounter mistreatment and perceive the learning environment less favorably are more likely to develop higher levels of exhaustion and disengagement, career regret, and less empathy.^12^ Furthermore, students with burnout are more prone to report engaging in unprofessional behaviors.^6^

Burnout prevalence increases as students advance in their training. A study conducted across three campuses in Kansas revealed that the year in training correlated with increased odds of burnout as well as symptoms of depression and fatigue.^13^ Another study focusing on medical students from Maryland, enrolled between 2010 to 2016, showed a marked rise in high emotional exhaustion throughout medical school years, from a prevalence of 9.4% among first-year students to a surge of 46.2% among fourth-year students.^14^ Similarly, the prevalence of high depersonalization increased from 8.6% among first-year students to 52.5% among fourth-year students.^14^

This trend is reflected in other parts of the world, such as Spain,^15^ or Mexico,^16^ indicating an increasing linear pattern in burnout rates that are notably higher in the later years than earlier years of medical education. This evidence underscores that burnout commences early in medical training and intensifies throughout careers in the medical field. Given that burnout and other emotional distress increase over the course of medical school, regardless of what school or campus the students attend, global interventions should be considered.

Gender, race, ethnicity, and potentially other variables can serve as significant risk factors contributing to BS among medical students. For example, the overall average burnout frequency appears to be slightly higher among females in comparison to males.^17^ Despite the importance of understanding these disparities, there is a notable lack of research specifically addressing race or ethnicity among medical student cohorts in the United States (Table 1).

**Table 1:** Burntout in medical students in the U.S.

Author	Year	Location	Prevalence	Size
Dyrbye^9^	2008	Multicenter	49.6%	4,287
Dyrbye^6^	2010	U.S.	52.80%	2,566
Dyrbye^8^	2014	National	55.9%	2,378
Ofei-Dodoo^13^	2019	Kansas	48%	379
Almutairi^10^	2022	U.S.	49.99%	14,320

Very few studies in the U.S. have analyzed race or ethnicity amongst their cohorts. We were very interested in exploring the situation for certain groups like Hispanic medical students, as it has been recently reported that medical students who are underrepresented in medicine face a significantly greater risk of experiencing burnout.^18^ Surprisingly, literature review revealed that there is very scarce data on burnout in Hispanic medical students. A study on wellness and resilience in fourth year medical students from Florida included only 16 out of 122 (13.1%) Hispanic students.^19^ Considering Florida's large Hispanic population, these figures notably underrepresent the demographic. Similarly, a larger study on wellness in medical students from 38 states included 80 out of 1,377 (5.8 %) Hispanic students.^20^ However, there exists extensive information on burnout in students from Latin America, which serves as the primary focus of this review.

## Literature search

An online search was conducted comprising the National Center for Biotechnology Information (NCBI), as well as Google search engine. No publication year limit was imposed.

Our first search aimed to obtain data on Burnout syndrome rates in Hispanic medical students in the U.S. The terms used were "Burnout," "Burnout syndrome," "medical students," "medical learners," "medical trainees," and "ethnicity," "race," "Hispanic," "Latin," "Latino," "Latinx." Since data was scarce, and the study took a direction to delve into burnout in Latin American medical students, this required the addition of terms "Latin America." The second search included studies that examined the variable of COVID-19, and the term "COVID-19" was added.

In order to avoid language restriction to only studies published in English, and since much information may come from Spanish-speaking Latin American countries a similar set of term in Spanish.

## Burnout in Latin America

BS is already a problem in medical professionals in most Latin American countries.^21^ The situation of medical students in Latin America, assessed in a pre-COVID-19 pandemic review, revealed again an escalating trend where higher study years corresponded with increased exhaustion levels.^22^ This aligns with established findings worldwide, suggesting that demands and stressors intensify as students’ progress through their medical training, leading to heightened burnout risk. This consistent prevalence shows there exists systemic challenges within medical education and practice that contribute to burnout.

The prevalence of BS in Latin American countries varies widely, ranging from 4.3 to 43.90% during pre-COVID-19 pandemic years (Table 2). Such variability may indicate both differences in the educational environment and the complex interplay of cultural, academic, and systemic factors contributing to burnout among students in this region. It is important to note that some of these studies included interns, because intern year is the final year of medical school in certain countries.^23^

**Table 2:** Burnout rates in medical students from Latin American countries

Author	Year	Country	Burnout	N
Asencio-Lopez^16^	2016	Mexico	27.80%	72
Villavicencio-Castro^23^	2016	Peru	34.20%	81
Rojas-Melgarejo^24^	2017	Paraguay	28%	61
Merchan-Galvis^25^	2018	Colombia	4.30%	161
Serrano^26^	2018	Colombia	14%	182
Carbonell^27^	2019	Colombia	31.70%	813
Gonzalez-Urbieta^28^	2020	Paraguay	43.90%	157
Ochoa^29^	2021	Argentina	29.44% (MS2) 32.40% (MS4)	87
Diaz-Flores^30^	2022	Mexico	69.87%	156

MS2 = second year medical students; MS4 = fourth year medical students.

Several studies provide valuable insights into burnout prevalence and its associations, highlighting the interrelation between burnout and mental health challenges. In Colombia, one study reported a of 4.3 % prevalence of burnout, with nearly half the students (47.8%) experiencing reduced personal realization.^25^ A following study highlighted the strong association between BS, depression (14% with burnout, 48% with depression), and feelings of financial inadequacy.^26^

In Argentina, a study indicated varying degrees of burnout among medical students across different study years, with second year students showing 55.55% mild and 44.44% moderate burnout, while fourth year medical students presented 50% mild, 40.90% moderate, and 9.09% profound burnout.^29^

In Paraguay, a study reported an overall BS prevalence in medical students of 28%, characterized by high levels of depersonalization (9.8%), emotional exhaustion (9.8%), and low levels of personal realization (16.4%).^24^ A later study reported a staggering 43.9% met the criteria for BS, alongside concerning statistics such as alcohol misuse (49%), high probability criteria for major depressive disorder (38.9%), and significant levels of suicidal ideation.^28^ These correlations underscore the multifaceted nature of burnout, emphasizing its impact on students’ psychological well-being and the urgency for holistic support systems within medical education.

The COVID-19 pandemic posed unique challenges for medical students. Few studies on medical student burnout related to COVID-19 are available. A small study from Brazil found that 28.2% of 209 medical students experienced burnout.^31^ The pandemic's disruption to educational routines and increased work demands likely exacerbated existing stressors.

Post-COVID-19 pandemic investigations in Mexico uncovered a prevalence of 69.87% for burnout, with emotional exhaustion being the most affected dimension, particularly among women (71% at medium and high levels). This report also found high rates of chronic diseases, depression and anxiety.^30^ These insights call for tailored interventions addressing not only burnout but also the interconnected mental health challenges faced by medical students in Latin America. The current scenario of the impact of COVID-19 on medical students which at this point constitute the new medical workforce is unknown in Latin America

## Associated depression, anxiety, and other comorbidities

The intersection of various psychiatric comorbidities may present a complex landscape that significantly impacts the emergence and exacerbation of BS in Latin American medical students. Generally speaking, BS is associated with a higher rate of substance abuse, poor interpersonal relations and occupational performance, and a lower perceived job satisfaction. Additionally, BS is tied to depression, anxiety, and suicidal ideation,^20^ with one cross-sectional study showing that more than one in ten medical students have experienced thoughts of suicide during their training.^9^ Studies show that burnout may persist beyond medical school, and is sometimes associated with psychiatric disorders and suicidal ideation.^31^ Meta-analyses have shown that approximately one-third of those students showed clinically relevant symptoms of depression,^32^ and anxiety.^33^ The prevalence of depressive symptoms has also been shown to increase across medical school years.^14,34^

When examining these psychiatric comorbidities in Latin America, it is important to mention that the average prevalence of suicidal ideation in Latin America is 13.85%, slightly below that observed in Europe and the United States.^35^

Reviewing the literature from Latin American countries, we found that in Honduras, major or minor depression was found in 31.57% medical students, with a remarkable 11.90% of those surveyed reporting thoughts of death or self-harm.^36^ Similarly, in Mexico, 21.82-35% of medical students reported anxiety while 16-20.2% reported depression.^37,38^ (Table 3). In El Salvador, 13.4 % of medical students reported having a previously diagnosed medical or psychiatric chronic condition; of these, 39% showed isolated anxiety, while 57% presented both anxiety and depression.^39^

**Table 3:** Rates of depression amongst Latin American medical students

Latin America				
Author	Year	Country	Depression	N
Terrones-Saldivar^37^	2010	Mexico	8%	75
Martinez^39^	2011	Colombia	27.10%	375
Guerrero-Lopez^40^	2013	Mexico	39.30%	455
Denis-Rodriguez^35^	2016	Mexico	8.60%	N/A
Villavicencio-Castro^23^	2016	Peru	35%	81
Serrano^26^	2018	Colombia	48%	182
Lemos^41^	2018	Colombia	52.60%	217
Medina-Guillen^36^	2020	Honduras	31.57%	380
Tadeo-Alvarez^42^	2019	Mexico	20.20%	203
Sandoval^43^	2019	Peru	24.30%	284
Leiva-Lopez^44^	2019	El Salvador	48.80%	N/A
Perez-Abreu^45^	2020	Cuba	86.40%	59
Rosales-Manrique^46^	2021	Peru	63.76%	145
Huarcaya-Victoria^47^	2021	Peru	74%	1238
Leiva-Nina^48^	2022	Peru	50%	110

Predictors of depression in Mexico included state of anxiety, stressors, and low socioeconomic level.^40^ The variables were associated with the symptomatic development of moderate-severe depression, anxiety and distress. Predictors of depression in Colombia were not having family economic stability, being in the first years of medical training, being female, and fearing delay and impairment of their medical training.^41^

In Colombia, pre-COVID-19 pandemic prevalence of depressive symptoms ranged from 27.1 to 56.2%, along with prevalences of alcohol use and abuse of 25.2% and 10.6%, respectively.^27,39,41^ Depression levels rose to an astonishing 80% by 2020.^49^ Anxiety was also high, ranging 48.3 to 59.9% in this country.^27,41^

Although the COVID-19 pandemic appears to have worsened the prevalence of anxiety and depression worldwide,^50,51^ with the values observed in Latin America being particularly high (Table 4). A recent multicenter study from Peru aiming to analyze the effect of the COVID-19 pandemic reported that depressive symptoms were at 74%, anxiety symptoms in 57%.^47^ In this country, during the COVID-19 pandemic, rates of moderate depression ranged 13.3- 50%, while anxiety was 28.5%.^43,46,48^ Furthermore, depression was associated with stress in this population.^50^ These pathologies occurred more frequently in the female sex, low socioeconomic levels, and in those who had clinical courses.^46,48^

**Table 4:** Anxiety rates among Latin American medical students

Latin America				
Author	Year	Country	Anxiety	N
Terrones-Saldivar^37^	2010	Mexico	69%	75
Reyes-Carmona^38^	2017	Mexico	21.82%	479
Lemos^41^	2018	Colombia	48.30%	217
Sandoval^43^	2019	Peru	28.50%	284
Carbonell^27^	2019	Colombia	59.90%	813
Perez-Abreu^45^	2020	Cuba	81.34%	209
Monterrosa-Castro^46^	2020	Colombia	43.60%	697
Huarcaya-Victoria^47^	2021	Peru	57%	1238
Leiva-Nina^48^	2022	Peru	85.46%	110

Anxiety and depressive symptoms being more frequent in women appears to be a common denominator in Latin America,^40,42,43^ and has been observed in other part of the world as well.^34^ Anxiety and emotion dysregulation have a significant direct influence on depression, coping skills, and alcohol consumption.^52^

The correlation between burnout and mental health disorders, compounded by the elevated prevalence of these disorders among medical students compared to the general population, presents a concerning issue that demands attention.

## Addressing medical student wellness

Wellness includes intellectual, emotional, physical, social, occupational, financial, environmental, and spiritual aspects. Students that feel less supported and uncomfortable with their social and daily environments express decreased satisfaction, sense of purpose, and financial status.^20^ Lower confidence and satisfaction with their medical education is associated with increased anxiety and depression.^20^

Medical students belonging to underrepresented minorities experience medical training more negatively, owing to structural racism and bias in classroom and health care delivery settings. These students also experience more discrimination episodes which translate to higher levels of exhaustion-related burnout.^18^

In Honduras, 93.7% of medical students declared that the career generated some degree of stress due to the academic load and demands.^36^ Reported risk factors for stress and emotional symptoms included being female, being in a basic cycle, not involved in extracurricular activities, low scores in problem solving, high aggression scores, and difficulties in expression and dealing with problems.^41^ Therefore, it is possible that inherent personality traits may be associated with developing BS during medical training.

High burn-out levels have been reported among medical students during their clerkships.^53^ Medical students’ negative perceptions of their colleagues’ work–life balance during their clinical rotations could be related to burn-out in clerkships. Witnessing separation of patient care or differences in patient care on the basis of insurance has been shown to contribute to cynicism and burnout.^54^

## What to do?

Resiliency may be an important protective factor for BS, along with being older, married, or having better academic performance.^20^ Family support for studying medicine is associated with lower burnout levels.^15^ Medical students living with a companion have decreased burn-out levels than did those living alone during their clerkships.^53^

Weekly participation in sport activities have been found as a protective factor for depression, anxiety, and stress.^44^ Having personal time after school, exercise or participate in physical activity for at least 30 minutes most days of the week, and being able to stop thinking about medical school after leaving for the day may be healthy habits for medical students, as higher resilience is associated with having the ability to stop thinking about school.^19^

Burnout in medical students is becoming highly prevalent, and significantly related to heavy workload and other factors of work life. Adequate changes of academic and organizational settings of medical curricula are needed to mitigate this serious problem. Although we did not find reports on successful intervention programs in Latin America, we should point out that such intervention needs to consider not only the socioeconomic context of medical students in their specific environment, but also the various factors that contribute to a high prevalence of burnout in such places. These should include (and not limit to) policies for mental health screening, support systems, curriculum changes, and student wellness programs. Taking into account the cultural context would have much to do with the success or failure of intended interventions.

## Data Availability

The data that support this study are available from the authors upon request.
